# Supplementary material for: MetaQTL: a package of new computational methods for the meta-analysis of QTL mapping experiments
Source: BMC Bioinformatics. 2007 Feb 8;8:49. doi: 10.1186/1471-2105-8-49 (PMC1808479; doi:10.1186/1471-2105-8-49)
Supplement: Additional File 7 — MetaQTL Package : jar file and tutorial. This Zip archive contains both the MetaQTL JAR file and the files of the tutorial. [file 1471-2105-8-49-S7.zip › org.inra.metaqtl/doc/org/metaqtl/main/MetaMain.html]

MetaMain


|  |  |  |
| --- | --- | --- |
| **Overview** \| **Package** \| **Class** \| **Use** \| **Tree** \| **Deprecated** \| **Index** \| **Help** | |  |
| **PREV CLASS**   **NEXT CLASS** | **FRAMES**    **NO FRAMES**     **All Classes** |  |
| SUMMARY: NESTED \| FIELD \| CONSTR \| METHOD | DETAIL: FIELD \| CONSTR \| METHOD |  |


---


## org.metaqtl.main Class MetaMain

```
java.lang.Object
  org.metaqtl.main.MetaMain
```

**Direct Known Subclasses:**: A2Xml, ConsMap, InfoMap, MapView, MetaDB, MMapView, MQTLView, QTLClust, QTLClustInfo, QTLModel, QTLProj, QTLTree, Xml2A

---

``` public abstract class MetaMain extends java.lang.Object ```

---

| **Field Summary** | |
| --- | --- |
| `protected  java.lang.Boolean` | `help` |
| `protected  CmdLineParser` | `parser` |
| `protected  java.lang.Boolean` | `verbose` |


**Constructor Summary**

`MetaMain()`


| **Method Summary** | |
| --- | --- |
| `static void` | `generalHelp()` |
| `static java.lang.String` | `generalUsage()` |
| `static Chromosome[]` | `getChromByNames(Chromosome[] chroms, java.lang.String[] chromNames)` |
| `static ChromGraph[]` | `getChromGraph(Chromosome[] chroms, boolean withQtl, int modeQtl, IBioOntology ontology, MetaQtlAnalysis treeAnalysis)` |
| `protected static MetaQtlModel` | `getCluster(java.lang.String cluFile)` |
| `static ChromCluster[]` | `getClusterByNames(ChromCluster[] clusters, java.lang.String[] chromNames)` |
| `protected static MetaDico` | `getDico(java.lang.String dicoFile)` |
| `protected static IBioLocus[]` | `getDubious(java.lang.String dubFile)` |
| `static IBioGenome` | `getMap(IBioGenome[] maps, java.lang.String mapName)` |
| `protected static IBioGenome` | `getMap(java.lang.String mapFile)` |
| `protected static IBioGenome[]` | `getMaps(java.lang.String qtlDir)` |
| `static java.util.Hashtable` | `getMrkToRm(java.io.File file)` |
| `protected static IBioOntology` | `getOntology(java.lang.String ontoF, char format)` |
| `static QtlPartition` | `getQtlPartition(Chromosome chromosome, IBioOntology ontology)` |
| `static QtlPartition` | `getQtlPartition(Chromosome chrom, MetaQtlAnalysis analysis, java.lang.String trait, int k, char tclust)` |
| `protected static MetaQtlAnalysis` | `getResult(java.lang.String resFile)` |
| `void` | `initCmdLineParser()` |
| `static java.lang.String[]` | `parseChromList(java.lang.String list)` |
| `void` | `parseCmdLine(java.lang.String[] args)` |
| `abstract  void` | `printHelp()` |
| `static void` | `printImgFormat(int indent)` |
| `static void` | `printLicense(java.lang.String program, java.lang.String version)` |
| `abstract  void` | `printUsage()` |
| `static void` | `removeLocus(IBioGenome[] maps, java.lang.String mrkRmFile)` |
| `static void` | `setParameter(java.lang.String parFile)` |
| `static void` | `synchChromGraphs(ChromGraph[] chromGraphs, java.util.Set partNames)` |
| `static void` | `writeImg(ChromGraph[] chroms, java.io.File file, java.lang.String imgFormat)` |
| `protected static void` | `writeMap(IBioGenome map, java.lang.String outFile)` |
| `protected static void` | `writeMap(IBioGenome genome, java.lang.String format, java.lang.String outFile)` |
| `static void` | `writePar(java.io.File file)` |

**Methods inherited from class java.lang.Object**

`clone, equals, finalize, getClass, hashCode, notify, notifyAll, toString, wait, wait, wait`

**Field Detail**

### parser

```
protected CmdLineParser parser
```

---


### verbose

```
protected java.lang.Boolean verbose
```

---


### help

```
protected java.lang.Boolean help
```


**Constructor Detail**

### MetaMain

```
public MetaMain()
```


**Method Detail**

### printUsage

```
public abstract void printUsage()
```

---


### printHelp

```
public abstract void printHelp()
```

---


### initCmdLineParser

```
public void initCmdLineParser()
```

---


### parseCmdLine

```
public void parseCmdLine(java.lang.String[] args)
```

---


### generalHelp

```
public static void generalHelp()
```

---


### generalUsage

```
public static java.lang.String generalUsage()
```

:   **Returns:**

---


### writeMap

```
protected static void writeMap(IBioGenome genome,
                               java.lang.String format,
                               java.lang.String outFile)
                        throws java.io.IOException
```

:   **Parameters:**: `genome` -: `format` -: `outFile` - **Throws:**: `java.io.IOException`

---


### writeMap

```
protected static void writeMap(IBioGenome map,
                               java.lang.String outFile)
                        throws java.io.IOException
```

:   **Parameters:**: `ap` -: `outFile` - **Throws:**: `java.io.IOException`

---


### getCluster

```
protected static MetaQtlModel getCluster(java.lang.String cluFile)
                                  throws java.io.IOException
```

:   **Parameters:**: `cluFile` - **Returns:**: **Throws:**: `java.io.IOException`

---


### getResult

```
protected static MetaQtlAnalysis getResult(java.lang.String resFile)
                                    throws java.io.IOException
```

:   **Parameters:**: `resFile` - **Returns:**: **Throws:**: `I`: `java.io.IOException`

---


### getMap

```
protected static IBioGenome getMap(java.lang.String mapFile)
                            throws java.io.IOException
```

:   **Parameters:**: `mapFile` - **Returns:**: **Throws:**: `java.io.IOException`

---


### getDubious

```
protected static IBioLocus[] getDubious(java.lang.String dubFile)
                                 throws java.io.IOException
```

:   **Parameters:**: `dubFile` - **Returns:**: **Throws:**: `java.io.IOException`

---


### getDico

```
protected static MetaDico getDico(java.lang.String dicoFile)
                           throws java.io.IOException
```

:   **Parameters:**: `dicoFile` - **Returns:**: **Throws:**: `java.io.IOException`

---


### getMaps

```
protected static IBioGenome[] getMaps(java.lang.String qtlDir)
                               throws java.io.IOException
```

:   **Parameters:**: `qtlDir` - **Returns:**: **Throws:**: `java.io.IOException`

---


### getOntology

```
protected static IBioOntology getOntology(java.lang.String ontoF,
                                          char format)
                                   throws java.io.IOException
```

:   **Parameters:**: `qtlF` - **Returns:**: **Throws:**: `java.io.IOException`

---


### printLicense

```
public static void printLicense(java.lang.String program,
                                java.lang.String version)
```

:   **Parameters:**: `string` -: `version` -

---


### getQtlPartition

```
public static QtlPartition getQtlPartition(Chromosome chromosome,
                                           IBioOntology ontology)
```

---


### getQtlPartition

```
public static QtlPartition getQtlPartition(Chromosome chrom,
                                           MetaQtlAnalysis analysis,
                                           java.lang.String trait,
                                           int k,
                                           char tclust)
```

---


### printImgFormat

```
public static void printImgFormat(int indent)
```

---


### setParameter

```
public static void setParameter(java.lang.String parFile)
                         throws java.io.IOException
```

:   **Parameters:**: `parFile` - **Throws:**: `java.io.IOException`

---


### parseChromList

```
public static java.lang.String[] parseChromList(java.lang.String list)
```

:   **Parameters:**: `list` - **Returns:**

---


### getChromByNames

```
public static Chromosome[] getChromByNames(Chromosome[] chroms,
                                           java.lang.String[] chromNames)
```

---


### getClusterByNames

```
public static ChromCluster[] getClusterByNames(ChromCluster[] clusters,
                                               java.lang.String[] chromNames)
```

---


### getChromGraph

```
public static ChromGraph[] getChromGraph(Chromosome[] chroms,
                                         boolean withQtl,
                                         int modeQtl,
                                         IBioOntology ontology,
                                         MetaQtlAnalysis treeAnalysis)
```

:   **Parameters:**: `chroms` -: `withQtl` - **Returns:**

---


### synchChromGraphs

```
public static void synchChromGraphs(ChromGraph[] chromGraphs,
                                    java.util.Set partNames)
```

:   **Parameters:**: `chromGraphs` -: `partNames` -

---


### writeImg

```
public static void writeImg(ChromGraph[] chroms,
                            java.io.File file,
                            java.lang.String imgFormat)
                     throws java.io.IOException
```

:   **Throws:**: `java.io.IOException`

---


### writePar

```
public static void writePar(java.io.File file)
                     throws java.io.IOException
```

:   **Parameters:**: `file` - **Throws:**: `java.io.IOException`

---


### getMrkToRm

```
public static java.util.Hashtable getMrkToRm(java.io.File file)
                                      throws java.io.IOException
```

:   **Parameters:**: `file` - **Returns:**: **Throws:**: `java.io.IOException`

---


### removeLocus

```
public static void removeLocus(IBioGenome[] maps,
                               java.lang.String mrkRmFile)
                        throws java.io.IOException
```

:   **Parameters:**: `maps` -: `mrkRmFile` - **Throws:**: `java.io.IOException`

---


### getMap

```
public static IBioGenome getMap(IBioGenome[] maps,
                                java.lang.String mapName)
```

:   **Parameters:**: `maps` -: `mapName` - **Returns:**


---


|  |  |  |
| --- | --- | --- |
| **Overview** \| **Package** \| **Class** \| **Use** \| **Tree** \| **Deprecated** \| **Index** \| **Help** | |  |
| **PREV CLASS**   **NEXT CLASS** | **FRAMES**    **NO FRAMES**     **All Classes** |  |
| SUMMARY: NESTED \| FIELD \| CONSTR \| METHOD | DETAIL: FIELD \| CONSTR \| METHOD |  |


---
